# Is Steatotic Liver Disease Related to Poor Outcome in COVID-19-Hospitalized Patients?

**DOI:** 10.3390/jcm13092687

**Published:** 2024-05-03

**Authors:** Fernanda Manhães Pozzobon, Ronir Raggio Luiz, Júlia Gomes Parente, Taísa Melo Guarilha, Maria Paula Raymundo Cunha Fontes, Renata de Mello Perez, Maria Chiara Chindamo

**Affiliations:** 1 Barra D’Or Hospital, Rede D’Or São Luiz, Rio de Janeiro 22775-002, RJ, Brazil; juliagparente@gmail.com (J.G.P.); taisamg@gmail.com (T.M.G.); mpfontes@outlook.com.br (M.P.R.C.F.); maria.chiara@barrador.com.br (M.C.C.); 2 Health Assistance Division, Federal Fluminense University (UFF), Niterói 24220-900, RJ, Brazil; 3 Institute for Collective Health Studies, Federal University of Rio de Janeiro (UFRJ), Rio de Janeiro 21941-598, RJ, Brazil; ronir@iesc.ufrj.br; 4 D’Or Institute for Research and Education (IDOR), Rio de Janeiro 22281-100, RJ, Brazil; renatamperez@gmail.com; 5 School of Medicine, Federal University of Rio de Janeiro (UFRJ), Rio de Janeiro 21044-020, RJ, Brazil

**Keywords:** steatotic liver disease, nonalcoholic fatty liver disease, COVID-19, SARS-CoV-2, outcome, mortality

## Abstract

**Background**: Steatotic liver disease (SLD) has been linked to more exacerbated inflammatory responses in various scenarios. The relationship between SLD and COVID-19 prognosis remains unclear. Our aim was to investigate the impact of SLD on the outcome of COVID-19. **Methods**: Patients hospitalized with confirmed COVID-19 and who underwent laboratory tests and chest CT scans were included. SLD was assessed by measuring the attenuation coefficient on CT scans. The relationship between SLD, the severity of COVID-19 clinical presentation and in-hospital mortality were assessed. **Results**: A total of 610 patients were included (mean age 62 ± 16 years, 64% male). The prevalence of SLD was 30%, and the overall in-hospital mortality rate was 19%. Patients with SLD were younger (58 ± 13 vs. 64 ± 16 years, *p* < 0.001) and had a higher BMI (32 ± 5 vs. 28 ± 4 kg/m^2^, *p* = 0.014). Admission AST values were higher in patients with SLD (82 ± 339 vs. 50 ± 37, *p* = 0.02), while D-dimer (1112 ± 2147 vs. 1959 ± 8509, *p* = 0.07), C-reactive protein (12 ± 9 vs. 11 ± 8, *p* = 0.27), ALT (67 ± 163 vs. 47 ± 90, *p* = 0.11), ALP (83 ± 52 vs. 102 ± 125, *p* = 0.27), and GGT (123 ± 125 vs. 104 ± 146, *p* = 0.61) did not significantly differ compared to patients without SLD. No difference was observed regarding lung parenchyma involvement >50% (20% vs. 17%, *p* = 0.25), hospital length of stay (14 ± 19 vs. 16 ± 23 days, *p* = 0.20), hemodialysis support (14% vs. 16%, *p* = 0.57), use of mechanical ventilation (20% vs. 20%, *p* = 0.96), and in-hospital mortality (17% vs. 20%, *p* = 0.40) when comparing patients with and without SLD. **Conclusions**: SLD showed no significant association with morbidity and mortality in patients with COVID-19.

## 1. Introduction

Metabolic syndrome, described by Reaven in 1988 [1], has been considered the disease of the century, with increasing prevalence rates worldwide that have reached 40% in some countries [2,3]. Closely related to obesity and diabetes mellitus (DM), the condition comprises a set of associated metabolic changes, such as hyperglycemia, dyslipidemia, insulin resistance, arterial hypertension, and visceral obesity, resulting in a pro-thrombotic and pro-inflammatory state accompanied by an increase in some cytokines and C-reactive protein (CRP) [4,5]. The chronic inflammatory state of patients with metabolic syndrome has been postulated to increase vulnerability to pro-inflammatory triggers of viral infections, which can worsen the outcomes of diseases caused by influenza virus and severe acute respiratory syndrome coronavirus 2 (SARS-CoV-2) [6].

Steatotic liver disease (SLD), an overarching umbrella term introduced in 2023 by a multinational consensus to encompass the various etiologies of steatosis [7,8], is currently the most common cause of chronic liver disease worldwide, with prevalence ranging from 13.5% in Africa to 31.8% in the Middle East and showing a substantial increase over the last decade in South America (30%) [9]. The main risk factors are obesity, type 2 diabetes mellitus, dyslipidemia, and metabolic syndrome. SLD has been related to more severe coronavirus disease 2019 (COVID-19), worse pneumonia progression, the need for hospitalization and mechanical ventilation (MV), and increased mortality risk [10,11]. However, studies on the influence of SLD on COVID-19 severity and outcome, most of which were performed in Asia, have assessed different populations and used heterogeneous methodologies for the SLD diagnosis, thus carrying several selection biases and yielding controversial results [11,12,13,14,15,16,17,18,19,20,21,22,23,24].

Considering the controversial findings about the influence of SLD on COVID-19 presentation, clinical course, and mortality, in addition to the scarcity of data on the Western population, it is important to assess the relationship between SLD and COVID-19 clinical outcome in that population, in which the incidence of metabolic syndrome and SLD is even higher than that in the Eastern population [25]. Understanding this relationship in developing countries with a high prevalence of obesity, metabolic syndrome, and SLD is of paramount importance for prioritizing vaccination and directing resources to hospitalized COVID-19 patients. This study was aimed at assessing the impact of SLD on the clinical outcomes of hospitalized patients with COVID-19 and to compare the COVID-19 clinical presentation and laboratory findings of patients with and without SLD.

## 2. Materials and Methods

### 2.1. Study Design and Population

This was a retrospective observational study that included adult patients diagnosed with COVID-19 and admitted to a private tertiary hospital in the city of Rio de Janeiro, Brazil, from March to December 2020. Patients with confirmed SARS-CoV-2 infection, laboratory tests, and chest or abdomen computed tomography (CT) imaging were included in this study. Patients who had undergone chest contrast-enhanced CT imaging, which hinders the assessment of liver steatosis, were excluded from this study, as were those with prolonged hospitalization and hospital-acquired SARS-CoV-2 infection.

### 2.2. Data Collection and Follow-up

SARS-CoV-2 infection was confirmed with viral RNA detection by use of the real-time reverse transcription-polymerase chain reaction (RT-PCR) technique on naso- and oropharyngeal swabs, targeting the N gene and RdRP gene regions of the coronavirus genome (Allplex™ assay, Seegene, Seoul, Republic of Korea).

The patients’ clinical, demographic, and laboratory data were retrieved from electronic medical records. The following clinical variables were assessed: sex; age; body mass index (BMI); comorbidities, such as systemic arterial hypertension (SAH), DM, chronic obstructive pulmonary disease (COPD), heart disease, chronic kidney failure, and obesity; extent of lung involvement; hospital length of stay; admission to the intensive care unit (ICU); need for hemodialysis (HD); use of MV; and in-hospital outcome (discharge or death). The laboratory data assessed were as follows: D-dimer (DD), CRP, alanine aminotransferase (ALT), aspartate aminotransferase (AST), alkaline phosphatase (ALP), and gamma-glutamyltransferase (GGT), measured by use of a colorimetric coupled enzyme assay; leukocyte and lymphocyte count, determined by use of flow cytometry and impedance; and international normalized ratio (INR), determined by use of a coagulometric method.

The following normal ranges for the laboratory tests were considered: DD ≤ 500 ng/mL; CRP ≤ 1.0 mg/dL; leukocyte count between 3500 and 10,500 cells/mm^3^; lymphocyte count between 900 and 2900 cells/mm^3^; AST ≤ 40 U/L; ALT ≤ 40 U/L; ALP ≤ 126 U/L; and GGT ≤ 73 U/L. The initial (on hospital admission) and peak (the highest levels during hospital stay) laboratory values were used for comparison in this study.

Overweight was defined as a BMI ≥ 25 and < 30 kg/m^2^, and obesity was defined as a BMI ≥ 30 kg/m^2^ [26].

**Figure 1:**
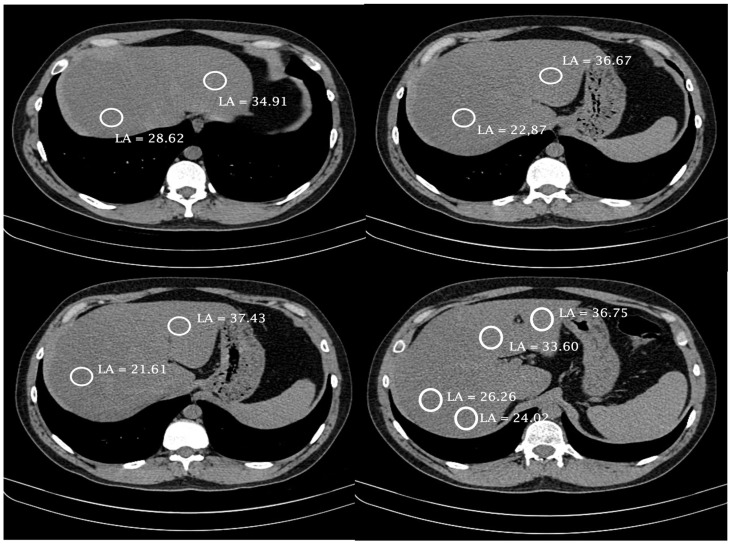
Computed tomography images and measurement sites of the attenuation coefficient in one of the patients evaluated in the study. LA, liver attenuation.

Liver steatosis was detected on chest or abdomen CT imaging and quantified by use of the liver attenuation coefficient measured in four sites of the liver parenchyma (two measures in the right lobe, one in the right-to-left lobe transition, and one in the left lobe) during the non-enhanced phase, as shown in Figure 1. The contrast is a confounding effect because it alters hepatic density, and there are no cut-off density value parameters that allow for characterizing steatosis in a post-contrast study. The most homogeneous areas of the liver parenchyma were chosen, avoiding vessels, the biliary tract, and focal liver lesions [27]. Later, the arithmetic mean of the four measures was calculated. Mean attenuation coefficient values ≤ 40 Hounsfield units were considered indicators of SLD [24,28].

The extent of lung involvement was assessed on chest CT imaging performed on admission and during hospitalization, when necessary. When more than one chest CT scan was performed, the one with the most extensive lung involvement of each patient was analyzed. The CT reports followed a structured pattern according to the Radiological Society of North America expert consensus document on reporting chest CT findings related to COVID-19 [29]. The CT scans were reviewed by a single radiologist who had no knowledge of the clinical data, and they were classified as follows: (a) typical CT imaging with lung parenchyma involvement of up to 25%, 25–50%, or greater than 50%; (b) atypical or indeterminate CT imaging; and (c) normal CT imaging [29].

### 2.3. Statistical Analysis

Continuous variables were reported as means, and discrete variables were reported as absolute values (n) and relative frequency (%). Comparisons between independent groups were evaluated by the Mann–Whitney and chi-square tests for quantitative and qualitative comparisons, respectively. The clinical and laboratory findings on the first day of hospitalization (hospital admission) were considered as baseline values, and the highest values of the entire hospitalization period were considered as peak values. The primary outcome assessed was in-hospital mortality. To analyze the factors independently associated with mortality, logistic regression analysis was used. The univariate logistic model and the multivariate logistic model with all variables were adjusted, as was the final model with all statistically significant variables. The analysis was performed using the SPSS package, version 29, 2022 (Property of IBM Corporation, Armonk, NY, USA). Significance level was determined when *p* ≤ 0.05 assuming two-tailed tests.

## 3. Results

**Figure 2:**
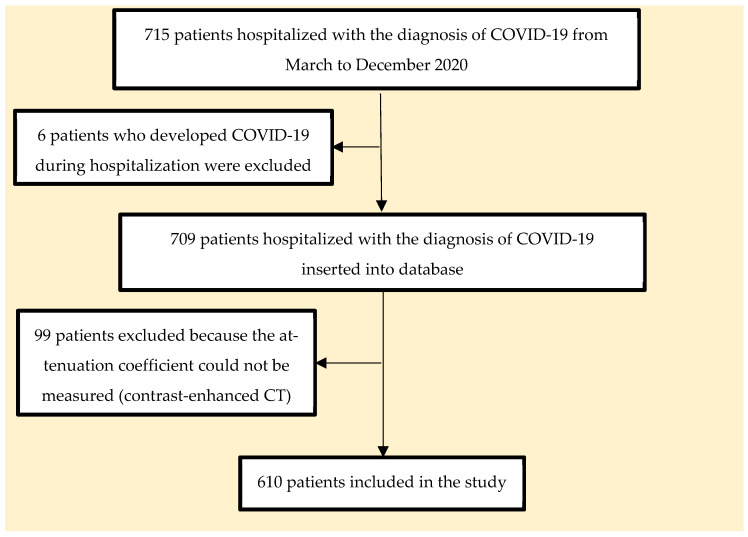
Flowchart of patients included in the study. CT, computed tomography.

This study selected 715 patients hospitalized with a diagnosis of COVID-19, of whom 6 who developed COVID-19 during hospitalization and 99 who underwent chest CT with venous contrast enhancement, which hindered the assessment of hepatic steatosis, were excluded, as shown in Figure 2.

This study, thus, included 610 patients with the following characteristics: mean age of 62 ± 16 years (20–101); 388 (64%) of the male sex; and 69% with two or more comorbidities. The comorbidities most frequently observed were SAH (52%), obesity (38%), and DM (30%). Of all patients, only 20% had a normal BMI. In the general sample, the prevalence of obesity was 38% (n = 231) and that of overweight was 42% (n = 256). The mean BMI was 29 kg/m^2^ (17–50).

Regarding clinical course, 69% of the patients were admitted to the ICU, 20% required MV, and 15% needed HD during their hospitalization.

Increases in the AST and ALT levels were observed in 56% and 40% of the patients on hospital admission and in 66% and 60% during hospitalization, respectively.

### 3.1. Steatotic Liver Disease

The prevalence of SLD was 30%. The patients were divided into two groups according to the presence/absence of SLD. Table 1 and Table 2 show the comparison between the groups with and without SLD. The group with SLD had a higher percentage of men, a lower mean age, and higher AST levels. The groups did not differ regarding the other variables assessed.

A higher percentage of patients with SLD had elevated AST and ALT levels on hospital admission as compared to patients without SLD [64% vs. 52% (*p* = 0.019) and 50% vs. 35% (*p* = 0.001), respectively]. The same was observed during hospitalization: 72% vs. 64% (*p* = 0.052) and 71% vs. 55% (*p* < 0.001), respectively.

Regarding the progression of the clinical and laboratory findings during hospitalization, the two groups were similar, except for the frequency of admission to the ICU, which was higher in the group with SLD (Table 2). The chest CT scan with the most extensive lung involvement of each patient during hospitalization was compared in the groups with and without SLD, and no significant difference was found between the groups.

### 3.2. Mortality

In this study, the mortality rate was 19.0% and was similar for men and women (18.3% vs. 20.3%; *p* = 0.56). In-hospital mortality did not significantly differ between the groups with and without SLD (17% vs. 20%; *p* = 0.40).

**Scheme 1:**
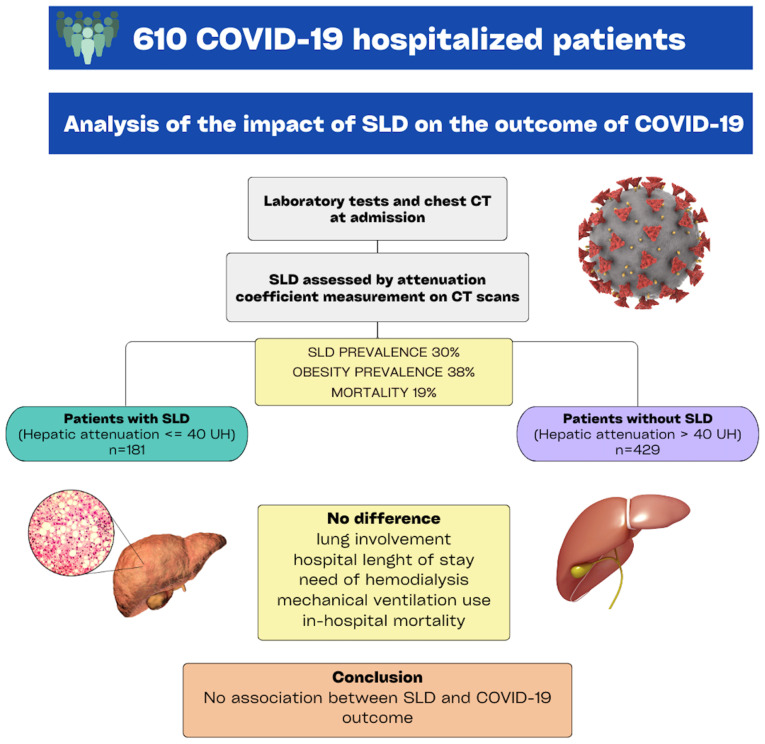
Visual summary illustrating the comparation between the groups with and without steatotic liver disease (SLD).

In the logistic regression analysis, the variables related to mortality were age (OR 1.10 [95% CI, 1.06–1.14; *p* < 0.001]), need for HD (OR 20.40 [95% CI, 7.13–58.41; *p* < 0.001]), need for MV (OR 38.83 [95% CI, 16.21–114.30; *p* < 0.001]), lung involvement > 50% (OR 6.23 [95% CI, 2.51–15.45; *p* < 0.001]), and hospital length of stay (OR 0.98 [95% CI, 0.96–0.99; *p* = 0.012]). The presence of SLD did not correlate with mortality, as shown in Table 3 and in Scheme 1.

## 4. Discussion

This study assessing a large cohort of Brazilian patients hospitalized with the diagnosis of COVID-19 and with a high prevalence of SLD found no association between SLD and COVID-19 clinical severity. The clinical courses of the groups with and without SLD were similar regarding the total hospital and ICU length of stays, need for MV and HD, and extent of lung involvement. In addition, no relationship between SLD and in-hospital mortality was found in this sample.

Obesity was initially considered a risk factor for unfavorable clinical course of disease in studies conducted at the beginning of the COVID-19 pandemic, a perception that resulted in the recommendation for higher clinical surveillance and early hospitalization of obese patients [30,31,32]. Dysregulation of the immune response to respiratory infections and impact on the ventilatory function of patients with COVID-19 would associate with higher morbidity and mortality among them [30,31]. However, some subsequent studies performed throughout the pandemic have shown the paradoxical effect of obesity, suggesting that patients with moderate obesity could be at lower risk for mortality, while the negative effects of obesity could be more marked only in the subgroup of severely obese patients (BMI > 40 kg/m^2^) [33,34].

Considering the significant relationship between obesity, metabolic syndrome, and SLD, as well as the existence of controversial results on how those clinical conditions influence the clinical course of SARS-CoV-2 infection, subsequent studies have aimed at defining the impact of SLD on COVID-19 outcome [6,11,12,13,16,17,18,19,20,21,22,23,24,35,36,37]. However, the clinical profiles of the populations studied, as well as the SLD prevalence and the SLD diagnostic methods used, differed. These discrepancies have hindered the interpretation of those studies’ results, evidencing the need for deeper analysis.

This study carried out a detailed assessment of SLD by calculating the attenuation coefficient on CT scans in four points of the liver parenchyma by a single radiologist, which reduced the interobserver and intraparenchymal variabilities. Our results showed SLD in 30% of the patients, similarly to the Latin American prevalence reported in previous studies [38,39]. The frequency of SLD in studies using the attenuation coefficient for its diagnosis has ranged from 16% to 38% [18,22,24,35,36,37], a variation that can be explained by differences in study methodology and the local prevalence of SLD.

In our study, the patients diagnosed with SLD had higher AST levels on hospital admission and a higher percentage of liver injury on hospital admission and during hospitalization as compared to patients without SLD. These data are in accordance with those reported by Huang et al. in the 2020 Chinese study involving 19 hospitals [12]. In addition, a higher frequency of ICU admission was observed in the group with SLD, which should be carefully analyzed, because it might reflect issues of bed management or a need for respiratory isolation and not only disease severity. Furthermore, the higher frequency of ICU admission could be related to a higher concern about a possible unfavorable clinical course because patients with SLD are more often obese, as shown in Table 1.

One of the challenges to the interpretation of the role played by SLD in the COVID-19 outcome is the heterogeneity of the methods used for diagnosing SLD. Several studies have applied non-invasive scores for this, such as the hepatic steatosis index (HSI) and the fibrosis-4 index for liver fibrosis (FIB-4), which include the levels of aminotransferases in their calculation formulae [11,13,14,15,19,21,23]. This might result in overestimating the prevalence of SLD, considering that COVID-19 is often associated with increased levels of aminotransferases [11,13,14,15,19,21,23]. A Mexican study by Velasquez et al., using the HSI, has found a 76% SLD prevalence, which is higher than that reported in the Mexican population [14]. In addition, it is worth noting that the indirect markers of fibrosis have not been validated in the COVID-19 population. Thus, we cannot claim that those changes resulted exclusively from liver fibrosis without considering the possible influence from the viral infection itself [35]. Furthermore, previous studies [40,41,42], including one by our research group [43], have shown that liver injury can be a marker of COVID-19 severity; thus, using diagnosis scores for SLD that include in their calculation formulae the levels of aminotransferases could misdiagnose more severe cases of COVID-19 as cases of SLD, which represents a selection bias.

The use of radiological methods for the diagnosis of SLD has been less explored in studies. Ji et al. [21], conducting one of the few studies using ultrasonography (US) to assess SLD, included 202 patients and diagnosed SLD with US and/or the HSI. In their study, liver injury was identified in 75% of the patients throughout hospitalization, and SLD was diagnosed in 38% of the patients, a higher percentage than that in Chinese studies, which could reflect overestimation by the HSI.

Regarding outcome, in our study, the overall in-hospital mortality was 19%, in line with some publications [11,44,45,46,47]. However, the observational cross-sectional study by Portela et al. [48] carried out from 2020 to 2022 in public, private, and philanthropic institutions of all Brazilian regions has shown a 32% COVID-19 in-patient mortality rate at the national level, higher than that found in our study. It is worth noting that, despite the clinical severity of our patients, with a significant ICU admission rate, 20% of whom were on MV and 15% required HD, our sample comprised cases exclusively from a private hospital in the Brazilian southeastern region, which is known to have better hospital and ICU structures as compared to those of public hospitals and of other Brazilian regions. Thus, these characteristics could justify the lower mortality in our case series as compared to that of the study by Portela et al. [48].

Few studies have investigated the impact of SLD on COVID-19 clinical presentation and outcome by using CT imaging for its diagnosis through the attenuation coefficient calculation [17,18,22,24,35,36,37]. Most of those studies have identified an association between SLD and COVID-19 severity, a finding that differs from ours. However, the populations and methodologies used were different from those of the present study [17,18,24,35,36,37]. Zhou-Y-J et al. [17], for example, have shown a correlation between the presence of SLD and a high severity of COVID-19. However, the population of that study comprised patients under the age of 60 years, thus, younger than ours, and no mortality data were reported. A Chinese case–control study [18] compared 587 patients hospitalized due to COVID-19 (cases) with 587 non-hospitalized patients who had undergone chest CT in the year preceding the pandemic due to other reasons (controls). That case–control study found a longer hospital length of stay and higher severity of lung involvement, but no relation to mortality, in the group with SLD as compared to the group without SLD. Another limitation was that both groups (cases and controls) underwent tests for the diagnosis of SLD in different occasions, which might have hindered the analysis of the results.

Targher et al. [35] compared patients with and without SLD, diagnosed by the use of CT imaging, and with different grades of liver fibrosis, assessed by the use of FIB-4. They found a higher COVID-19 severity in patients with SLD in the subgroup of intermediate or high FIB-4. However, the use of non-invasive markers, involving aminotransferases in their calculation formulae, might have influenced those findings. Similarly, an Israel study with 71 patients has shown the relationship of SLD with the severity of COVID-19 clinical presentation; however, the methodology used for the diagnosis of SLD was not homogeneous, with the use of a CT scan in 59% of the patients and medical documentation in 41% of the cases [37]. Another Chinese study, conducted by Gao et al. [36], screened SLD using computed tomography and assessed the association between circulating interleukin-6 levels and SLD at hospital admission in relation to the risk of severe COVID-19. The study concluded that patients with SLD and elevated serum IL-6 levels were at higher risk of experiencing severe illness from COVID-19, highlighting the connection between the inflammatory profile of SLD and the severity of COVID-19 clinical presentation.

Differently from the studies reporting an association between SLD and a higher clinical severity of COVID-19 and in line with our findings, Nath et al., in a study assessing 3983 patients in India with a methodology similar to ours, found no association between SLD and worse clinical course or higher mortality [22]. However, it is worth noting that despite the substantial size of the study by Nath et al. [22], it cannot be directly compared with ours due to differences across key characteristics, such as the lower prevalence of SLD (20%) and low mortality rate (6.4%), suggesting that study assessed a group of less-severely-ill patients.

Therefore, previous studies using the same SLD diagnostic method (CT scan) as ours had methodological flaws or assessed populations with a low prevalence of SLD and/or lower clinical severity. Our study pioneered the assessment of the relationship between SLD and the outcome of patients with COVID-19 in Brazil. Our study comprised a large cohort of patients with more severe clinical characteristics, such as a mean age over 60 years and two or three comorbidities. SLD was assessed by the use of a CT scan, a well-established and more reliable method than serum markers, with higher sensitivity and specificity, as compared to the US, for the diagnosis of SLD [28,49,50,51]. Thus, in a population with a high prevalence of SLD and more severe clinical findings, there was no association between SLD, worse clinical course, and mortality.

This study has some limitations. One limitation of our study was its retrospective design, which prevented the addition of new variables to the database across the follow-up period. Additionally, the fact that it was conducted in a private center may hinder the extrapolation of the results to the general population. Another important issue was the use of the BMI as reference to define obesity because that variable can be influenced by non-measurable factors, such as fluid retention and sarcopenia, not reflecting the grade of body and visceral fat accumulation.

## 5. Conclusions

In conclusion, after conducting an in-depth analysis of a large sample of severe COVID-19 patients with a notably high prevalence of SLD and employing a diagnostic method characterized by low variability, our findings revealed that the presence of SLD did not exhibit an impact on the overall outcomes of these patients.

## Figures and Tables

**Table 1:** Comparison of clinical and laboratory findings on hospital admission of patients with and without SLD. SLD, steatotic liver disease; COPD, chronic obstructive pulmonary disease; BMI, body mass index; CRP, C-reactive protein; AST, aspartate aminotransferase; ALT, alanine aminotransferase; ALP, alkaline phosphatase; GGT, gamma-glutamyltransferase; INR, international normalized ratio.

	Total (n = 610)	With SLD (n = 181)	Without SLD (n = 429)	*p*
Male sex (%)	388 (64%)	139 (76%)	249 (58%)	<0.001
Age (years)	62 ± 16	58 ± 13	64 ± 16	<0.001
Arterial hypertension (%)	317 (52%)	99 (55%)	218 (51%)	0.25
Diabetes mellitus (%)	183 (30%)	63 (35%)	120 (28%)	0.08
COPD (%)	29 (5%)	8 (4%)	21 (5%)	0.80
Heart disease (%)	89 (15%)	22 (12%)	67 (16%)	0.26
Kidney failure (%)	40 (7%)	8 (4%)	32 (7%)	0.16
Obesity (%)	231 (38%)	107 (59%)	124 (29%)	<0.001
BMI (kg/m^2^)	29 ± 5	32 ± 5	28 ± 4	0.014
D-dimer (ng/mL)	1696 ± 7186	1112 ± 2147	1959 ± 8509	0.07
CRP (mg/dL)	11 ± 8.7	12 ± 9	11 ± 8	0.27
Leukocytes (/mm^3^)	6972 ± 3337	7156 ± 2856	6894 ± 3521	0.11
Lymphocytes (/mm^3^)	1212 ± 1745	1182 ± 501	1224 ± 2059	0.21
AST (U/L)	59 ± 191	82 ± 339	50 ± 37	0.02
ALT (U/L)	53 ± 118	67 ±163	47 ± 90	0.11
ALP (U/L)	96 ± 107	83 ± 52	102 ± 125	0.27
GGT (U/L)	110 ± 139	123 ± 125	104 ± 146	0.61
INR	1.14 ± 0.36	1.16 ± 0.5	1.13 ± 0.2	0.60

**Table 2:** Comparison of clinical and ‘peak’ laboratory findings of patients with and without SLD. SLD, steatotic liver disease; ICU, intensive care unit; CT, computed tomography; CRP, C-reactive protein; AST, aspartate aminotransferase; ALT, alanine aminotransferase; ALP, alkaline phosphatase; GGT, gamma-glutamyltransferase.

		Total (n = 610)	With SLD (n = 181)	Without SLD (n = 429)	*p*
Hospital length of stay (days)		16 ± 22	14 ± 19	16 ± 23	0.20
ICU admission (n, %)		420 (69%)	137 (76%)	283 (66%)	0.01
ICU length of stay (days)		13 ± 17	12 ± 15	14 ± 18	0.16
Hemodialysis (n, %)		92 (15%)	25 (14%)	67 (16%)	0.57
Mechanical ventilation (n, %)		122 (20%)	36 (20%)	86 (20%)	0.96
Mortality (n, %)		116 (19%)	31 (17%)	85 (20%)	0.40
D-dimer ≥ 500 ng/mL (%)		87%	87%	88%	0.73
CRP (mg/dL)		7 ± 9	20 ± 13	19 ± 13	0.98
AST (U/L)		902 ± 3600	266 ± 1388	319 ± 1970	0.60
ALT (U/L)		385 ± 1567	155 ± 495	163 ± 880	0.67
ALP (U/L)		167 ± 127	114 ± 106	140 ± 137	0.18
GGT (U/L)		190 ± 261	200 ± 298	186 ± 242	0.76
Chest CT	Lung involvement < 25%, or normal or atypical findings	225 (37%)	58 (32%)	167 (39%)	0.25
	Lung involvement of 25–50%	277 (45%)	87 (48%)	190 (44%)	
	Lung involvement > 50%	108 (18%)	36 (20%)	72 (17%)	

**Table 3:** Variables associated with mortality in logistic regression analysis. SLD, steatotic liver disease; COPD, chronic obstructive pulmonary disease; CI, confidence interval; AST, aspartate aminotransferase; ALT, alanine aminotransferase; HD, hemodialysis; ICU, intensive care unit.

Characteristics	Univariate Logistic Regression Model		Multivariate Logistic Regression Model		Final Multivariate Logistic Regression Model		
	OR	*p*-Value	OR	*p*-Value	OR	*p*-Value	95% CI
Sex	0.88	0.56	0.52	0.19			
Age	1.07	<0.001	1.13	<0.001	1.10	<0.001	1.06–1.14
SLD	0.83	0.43	1.16	0.81	1.25	0.65	0.48–3.27
Arterial hypertension	2.08	<0.001	0.42	0.14			
Diabetes mellitus	1.77	0.008	0.91	0.86			
Obesity	0.94	0.77	1.93	0.21			
COPD	3.76	<0.001	0.65	0.58			
Heart disease	2.41	<0.001	0.74	0.62			
Kidney failure	3.13	<0.001	1.65	0.49			
>2 comorbidities	5.95	<0.001	1.46	0.63			
Peak D-dimer ≥ 500 ng/mL	19.54	0.003	3.73	0.61			
Peak AST ≥ 40 U/L	5.96	<0.001	3.25	0.08			
Peak ALT ≥ 40 U/L	2.63	<0.001	1.28	0.68			
Need for HD	62.92	<0.001	27.24	<0.001	20.40	<0.001	7.13–58.41
Mechanical ventilation	119.00	<0.001	27.87	<0.001	38.83	<0.001	16.21–114.30
Lung involvement > 50%	6.00	<0.001	6.05	0.001	6.23	<0.001	2.51–15.45
ICU admission	13.33	<0.001	1.58	0.53			
Hospital length of stay	1.03	<0.001	0.98	0.007	0.98	0.012	0.96–0.99

## Data Availability

The raw data supporting the conclusions of this article will be made available by the authors without undue reservation.
